# Perceived Balance Problem Associated with Fall Prevention Behavior Among Hospitalized Older Adults

**DOI:** 10.1093/geroni/igaf122.118

**Published:** 2025-12-31

**Authors:** Hanne Dolan, Keenan Pituch, David Coon

**Affiliations:** Arizona State University, Phoenix, Arizona, United States; Arizona State University, Phoenix, Arizona, United States; Arizona State University, Phoenix, Arizona, United States

## Abstract

Inpatient falls among older adults remain a relentless problem. Older adults’ engagement in fall prevention is imperative, yet research indicates that up to 82% of hospitalized older adults do not perceive themselves as at risk for falling in the hospital despite being identified as such by nursing assessment. Recent studies found that older adults may prefer the term ‘balance problem’ as opposed to ‘fall risk.’ Using the Health Belief Model as its theoretical framework, the purpose of this study was to explore the interrelationships among perceived balance problem, balance confidence, fear of falling (FOF), falls history, and fall prevention behavior among hospitalized older adults. A cross-sectional analysis was conducted using survey data collected among 70 rurally hospitalized older adults (F = 57.1%, Mage = 77.8; SD = 8.7). A multivariable regression analysis found that older adults who indicated a balance problem reported greater fall prevention behavior (b = .41; SE = .16; p = .014; rsemi-partial = .23). No other variable in the model was independently associated with fall prevention behavior, including balance confidence, FOF, falls history, health factor- and socio-demographic variables. Hospitalized older adults’ fall prevention behavior was positively associated with their perceived balance problem. Our findings also align with research showing that self-reported falls was associated with having a balance problem. This suggests that assessing hospitalized older adults’ perceptions of their own balance would be an important addition to inpatient fall risk assessments and may be highly important in reframing the topic of fall prevention among older adults.

